# Cortical inhibition and activation during sensorimotor tasks in an aquatic environment: a pilot EEG study based on expert-novice paradigm

**DOI:** 10.3389/fspor.2026.1660332

**Published:** 2026-02-10

**Authors:** Jun-Xiong Li, Xiao-Ya Qin, Wei Huang, Xin-Wen Zhang, Shi-Chun Bao, Yu Liu

**Affiliations:** 1 School of Exercise and Health, Shanghai University of Sport, Shanghai, China; 2 Key Laboratory of Exercise and Health Sciences of Ministry of Education, Shanghai University of Sport, Shanghai, China; 3 National Innovation Center for Advanced Medical Devices, Shenzhen, China; 4 School of Athletic Performance, Shanghai University of Sport, Shanghai, China; 5 Shenzhen University of Advanced Technology, Shenzhen, China

**Keywords:** aquatic environment, beta spectrum, cortical inhibition, mean frequency, power, sensorimotor rhythm (SMR)

## Abstract

**Purpose:**

This study aimed to investigate cortical inhibition and activation during sensorimotor task performance in an aquatic environment using an expert-novice paradigm (national-level athletes vs. non-athlete controls).

**Methods:**

Twelve national-level athletes and twelve age- and gender-matched controls completed a repetitive elbow flexion-extension task under both aquatic and terrestrial conditions. 64-channel EEG data were collected to measure sensorimotor rhythms (SMR) power at the Cz electrode, as well as the mean frequency (MF) and task-to-baseline power ratios of theta, alpha, and beta bands across the frontal, frontal-central, central, central-parietal, and parietal regions. Both within-group and between-group comparisons were performed.

**Results:**

The main results showed that the swimmer group exhibited a significant reduction in SMR power during an aquatic task, while no significant change was observed in a terrestrial task. In contrast, the control group showed significant reductions in SMR power under both conditions. In beta-band activity, both groups showed significantly increased MF in task conditions. Task-related beta power in both groups remained broadly comparable to the resting baseline, with no obvious decrease. In attention, the control group showed a slight increase in MF and task-related beta power during the aquatic task compared to the terrestrial condition, whereas the swimmer group showed comparable or slightly lower MF and task-related power in the aquatic environment.

**Conclusion:**

These findings suggest that, through long-term training, swimmers develop enhanced sensorimotor adaptation during movement in aquatic environments. This adaptation appears to involve environment-specific cortical activation patterns, which may further facilitate motor execution in water.

## Highlights

1. Swimmers demonstrated a significant decrease in SMR power during aquatic tasks only, while controls exhibited significant reductions under both aquatic and terrestrial conditions.
2. Beta-band power remained comparable to resting baseline during continuous elbow flexion–extension, potentially reflecting the combined effects of post-movement beta rebound (PMBR) and task-related cortical beta desynchronization.
3. In the control group, beta-band MF and power were obvious lower during the terrestrial task than during the aquatic task, suggesting that stopping movement under aquatic conditions demands greater neural processing

## 1 Introduction

In recent years, an increasing number of EEG studies have moved beyond the traditional paradigm of “minimal behavior” and shifted their focus toward cortical activity during motor tasks in real-life situations (1). For instance, investigating cognitive functions in gait control and fall prevention has been shown to facilitate the development of biomarkers for predicting early fall risk in Parkinson's disease (2, 3). Similarly, examining the differences in cortical rhythms between active and passive walking during robot-assisted gait offers direct neural evidence of whether patients are exerting voluntary effort (4). In the field of sport science, mobile EEG recordings conducted under swimming-pool conditions have revealed plausible modulations in cortical activation before and after turns (5). Taken together, these findings highlight that moving from laboratory-based interventions to real-world assessments of dynamic person–task–environment interactions is crucial for deepening our understanding of how cognitive and neural mechanisms support motor performance (6, 7).

The expert–novice paradigm has long extensively utilized to investigate the adaptive changes in the structure and function of the nervous system induced by prolonged training, aiming to elucidate the central neural mechanisms underlying exceptional behavioral performance (8–11). Professional pianists exhibited markedly reduced alpha-band power during the execution of intricate key-pressing movements in contrast to simpler tasks, while non-pianists displayed consistent alpha-band power levels across all tasks (12). Similarly, highly skilled athletes in sports exhibited a state of cortical “calm and focus” by suppressing task-irrelevant neural activity during motor execution (13–16). This cortical inhibitory characteristic has been conceptualized as psychomotor efficiency, which refers to superior motor performance enabled by refined neural processes (17). Overall, compared with non-athletes, the neurodynamic characteristics of expert performers in sports tasks primarily involve two aspects: (1) functional activation of task-related processes and (2) inhibition of task-irrelevant processes (14, 18).

Electroencephalography (EEG), due to its high temporal resolution and relatively low cost, has become a widely used tool for investigating cortical activation and inhibition (19). Previous research has associated theta-band oscillations (4–8 Hz) with cognitive control and attentional regulation, often showing increased power during attention-demanding or working memory tasks (20). Alpha-band oscillations (8–12 Hz), predominantly observed during resting states, demonstrate event-related desynchronization (ERD) during motor task performance, signifying task-specific cortical engagement and neural processing (21). Conversely, alpha power increases in task-irrelevant cortical regions are believed to indicate inhibition of distracting information (22). Beta oscillations (13–30 Hz), which are prominent during rest, typically show significant power suppression during movement execution, suggesting the release of motor inhibition (23). Gamma-band activity (>30 Hz) reflects high-frequency synchronization among local neural populations and is often enhanced during fine motor control and focused attention, indicating strong cortical engagement (24). Moreover, trained athletes demonstrate distinctive EEG patterns that differentiate them from untrained individuals. Elite athletes show reduced alpha ERD during motor tasks, indicating more efficient neural processing (22, 25) and better ability to suppress irrelevant brain activity to sustain attention (26). However, stronger alpha desynchronization may occur during complex motor tasks to support optimal performance (27).

In competitive swimming, increasing speed relies on reducing drag and enhancing propulsive force, which requires swimmers to adjust limb positions rapidly and precisely in the water (28). Proprioception supports limb position control, but hydrodynamic resistance and buoyancy can disturb afferent proprioceptive signals (29, 30). In previous research, elite swimmers demonstrate superior proprioceptive acuity and motor stability, outperforming non-athletes in distance perception and trajectory estimation tasks (31, 32). Building on evidences that long-term training improves athletes' sensorimotor abilities, whether long-term training allows swimmers to develop superior sensorimotor function in water remains an important question.

To bridge this research gap, we employed an experimental paradigm adapted from previous work (32), in which swimmers and non-athletes executed identical sensorimotor tasks in aquatic and non-aquatic environments, simultaneously recording their EEG signals, aiming to investigate the impact of training and environmental factors on sensorimotor cortical activity. Drawing on findings from prior studies (33, 34), we hypothesized that, compared with individuals without systematic swimming training, swimmers would exhibit superior sensorimotor adaptation in aquatic environments. To test the hypothesis, we performed a comprehensive electrophysiological analysis using EEG-derived indices, including sensorimotor rhythm (SMR; 12–15 Hz) power and the mean frequency (MF) and power spectra of theta, alpha, and beta bands. SMR rhythms, localized in the sensorimotor cortex, reflect neural resource allocation during fine motor tasks (35, 36); while MF quantifies the intensity of cortical firing near the electrode and is associated with both energy expenditure and functional activation within a given frequency band (37, 38). During motor tasks, changes in alpha- and beta-band EEG power are often interpreted as indices of large-scale cortical neuronal synchronization and their involvement in active inhibitory control (39, 40). We speculate that long-term swim training enhances cortical inhibition during sensorimotor performance in an aquatic environment, which would manifest as reduced SMR power and increased beta-band MF and power spectra. This study focuses on motor control in aquatic environments, and its findings may provide valuable insights for developing EEG-based neurofeedback protocols tailored to swimmers.

## 2 Materials and methods

### 2.1 Participants

In line with previous studies employing similar paradigms (32), we specified an expected effect size of f = 0.30 and a desired statistical power of 0.80 to determine the required sample size. Twenty-nine right-handed participants were recruited in the study, with fifteen assigned to the swimming group (SG) and fourteen to the control group (CG), all without a prior history of upper extremity or severe musculoskeletal injury. After exclusion of participants with incomplete testing, 24 participants were included in the final analysis (12 in SG, 12 in CG). The general information for each group is shown in Table 1. The training background information for the SG is shown in Table 2. Participants were recruited from Shanghai Sport University, with swimmers being members of the university's competitive swimming team and having competed at national levels. Mean sport-specific training age is 11.6 years. The performance level of the swimmers was assessed using FINA points, calculated based on their fastest records (http://www.fina.org/content/fina-points). The control group comprised college students with no formal swimming training. The study was approved by the Ethics Committee of Shanghai Sport University. All experiments were conducted in strict accordance to the ethical guidelines outlined in the Declaration of Helsinki, with comprehensive informed consent obtained from all participants.

**Table 1:** General characteristics of subjects.

Subjects	SG (*n* = 12, m7, f5)	CG (*n* = 12, m6, f6)
Age(year)	20 ± 1.13	21.25 ± 1.82
Height(cm)	175.19 ± 8.06	167.99 ± 8.91
Weight(kg)	70.36 ± 11.91	58.71 ± 10.77

General characteristics of subjects of swimmer group (SG) and control group (CG).

**Table 2:** Competitive level of swimmer group.

No.	Sex	Style	Sport-specific training age (year)	Competitive level (FINA point）
1	m	IM	7.5	589
2	f	IM	9.6	655
3	m	BA	13.7	621
4	f	FR	12.3	844
5	f	FR	10	638
6	m	FR	13.2	547
7	m	FR	6.8	690
8	f	FR	13.5	593
9	f	FR	13.8	640
10	m	BA	15.2	765
11	m	FR	10	667
12	m	BU	13.8	674

m, male; f, female; BU, butterfly stroke; BA, back stroke; FR, freestyle; IM, individual medley.

### 2.2 Experimental protocol

**Figure 1:**
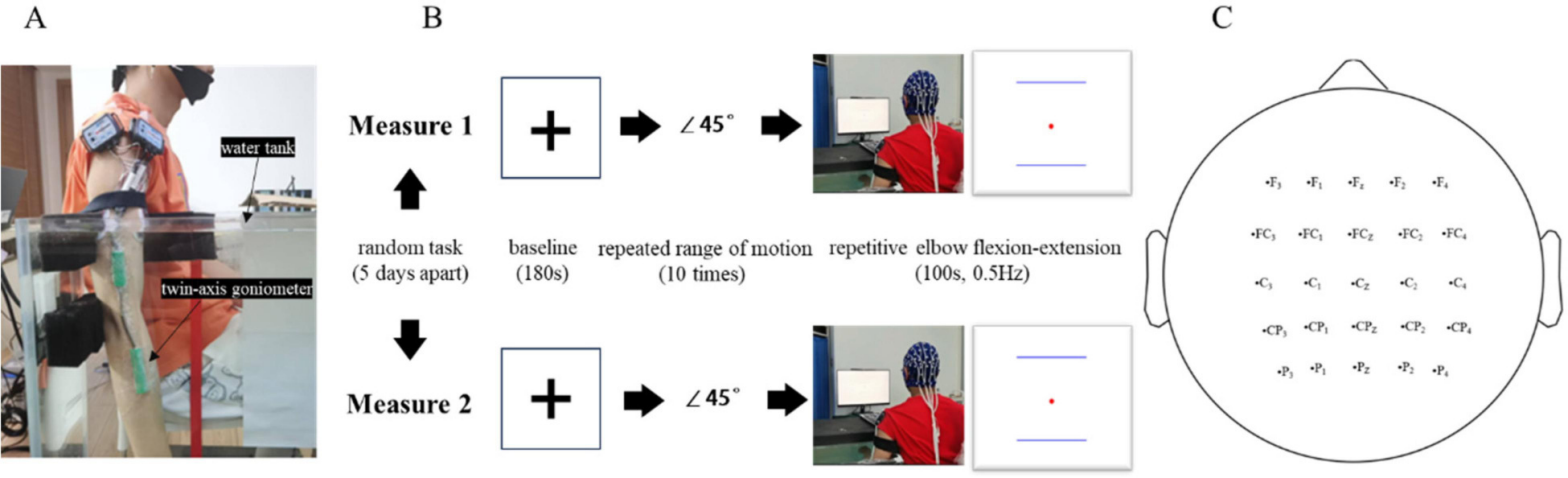
Task posture, experimental procedure, and EEG electrode locations. **(A)** Task posture. **(B)** Experimental procedure. **(C)** EEG electrode locations.

The participants underwent two experimental sessions, wherein they were randomly allocated to execute elbow flexion and extension movements in aquatic and terrestrial environments. EEG signals were continuously recorded during these sessions for subsequent neurophysiological analysis (Figure 1B).

#### 2.2.1 Motor tasks

During the experiments, participants were positioned in an upright, relaxed seated posture (Figure 1A). The test arm was inserted into either a water tank or an empty container (70cm × 45cm × 70 cm, identical in size), with the upper arm maintained in a strictly vertical orientation. Both left and right arms were separated tested in a randomized order. During the aquatic condition (water tank), the arm was submerged in the tank such that the lateral epicondyle of the humerus was positioned 10 cm below the water surface. Throughout elbow flexion and extension movements, the hand was required to remain fully submerged. In the terrestrial condition (empty tank), a barrier was placed 10 cm above the lateral epicondyle of the humerus to match the immersion depth in the aquatic condition, and participants were instructed to avoid contacting the barrier during the movement.

The participant sat 80 cm in front of the display screen. Prior to the commencement of the test, participants were guided through familiarization with elbow flexion and extension angles using verbal instructions from the experimenter. The angle of flexion and extension was recorded and displayed using a twin-axis goniometer (Biopac Systems, Inc., Goleta, CA, USA) with a sampling rate of 1,000 Hz. The experimental procedure involved ten consecutive elbow flexions and extensions cycle, each performed about 4 s and targeting a precise 45 ° range of motion. At the experiment's onset, a red ball vibrating up and down at 0.5 Hz appeared on the screen, and the subjects were instructed to follow the vibration frequency of the red ball as closely as possible while reproducing the flexion and extension angles that had been memorized previously. The total flexion and extension task was 100 s, with the entire test sequence controlled by E-prime 3.0 (3.0.3.80, Psychology Software Tools, Sharpsburg, PA).

#### 2.2.2 EEG data acquisition

EEG signals were recorded utilizing a 64-channel EEG system (Neuroscan, Germany) and digitized at a sampling rate of 1,000 Hz, adhering to the standardized international 10-10 electrode placement protocol. The reference electrode was placed between the Cz and CPz channels, and the impedance of all electrodes was maintained below 5 k*Ω*. Prior to the commencement of formal testing, participants were presented with a central cross mark (Figure 1A) on the monitor screen and instructed to maintain visual fixation during a three-minute resting-state EEG recording. Triggers were transmitted via E-Prime 3.0 to synchronize EEG recordings with behavioral motor tasks.

The preprocessing of the EEG data was conducted using EEGLAB 2024, a MATLAB-based open-source toolbox (41). To enhance signal quality and reduce potential artifacts, the middle segment (10th to 90th second) of the 100-second recording was selected for analysis. In subsequent analyses, only the channels related to motor and sensorimotor function were focused on. The examined channels were distributed across the following cortical regions: frontal (F_3_/F_1_/F_Z_/F_2_/F_4_), frontal-central (FC_3_/FC_1_/FC_Z_/FC_2_/FC_4_), central (C_3_/C_1_/C_Z_/C_2_/C_4_), central-parietal (CP_3_/CP_1_/CP_Z_/CP_2_/CP_4_), and parietal (P_3_/P_1_/P_Z_/P_2_/P_4_) regions in Figure 1C.

### 2.3 EEG processing

An offline band-pass filter of 1–40 Hz was applied. Independent component analysis (ICA) was performed using MATLAB-based EEGLAB toolbox with default parameters to remove electrooculogram (EOG) and electromyogram (EMG) components. EEG Data were re-referenced using the reference electrode standardization technique (REST) (42). Multitaper spectral estimation was used for power spectra of theta (4–8 Hz), alpha (8–12 Hz), beta (13–30 Hz), and SMR (12–15 Hz) waves with the FieldTrip toolbox (43). The data were segmented into 2-second epochs, with all available trials included in the analysis. The Fast Fourier Transform (FFT) was applied using the ft_freqanalysis function in Fieldtrip toolbox with the following settings: a 3 Hz smoothing frequency, DPSS tapers, and zero-padding to the next power of two for optimal frequency resolution. The power in the specified frequency band was averaged across trials. The power ratio was defined as the power in the task condition divided by that in the rest condition. The resulting frequency data were then stored for further statistical analysis. For MF, the formula is as follows (44), where P(f) was defined as power spectrum. *I* and *J* were the lower and upper limits of the corresponding frequency band.

$$\bar{\;f}=\frac{\sum_{\;f=i}^{j}\;(P(f)\times f)}{\sum_{\;f=i}^{j}\;(P(f))}$$

The percentage deviation between task and rest states was calculated using the formula Δ% = [(*T* value—*R* value)/*R* value] × 100, where *T* value represents the EEG eigenvalues during the task condition, and *R* value denotes the EEG eigenvalues during the rest state. The mean frequency for each cortical region was calculated as the average of all channels within that region. To characterize the overall trend, EEG data from the left and right elbow tasks were averaged prior to statistical analysis. The Schematic overview of the EEG data-processing and the result of power spectrum are provided in the Supplementary Materials (Supplementary Figures S1–S3).

### 2.4 Statistical analysis

The statistical analysis was conducted using SPSS 27.0 software, and descriptive statistics were performed on the raw data. Outliers in the raw data were defined as data points exceeding three times the interquartile range, with approximately 2.2% of the total data being replaced. The Shapiro–Wilk test was employed to assess the normality of the data. In this study, the testing time was the within-group factor (rest, terrestrial, aquatic), and the training experience was the between-group variable (SG, CG). For normally distributed data, analysis of variance (ANOVA) was used for comparisons of factors within groups, and independent *t*-tests were used for comparisons of factors between groups in the same condition. For non-normally distributed factors within groups, non-parametric statistical analysis was performed using nonparametric factorial ANOVA with aligned rank-transformed (ART) values. ART is a modification of the rank transform procedure by aligning data to strip the interaction effect from the main effects, as well as the main effects from each other and the interaction, and then ranking, factorial ANOVA is possible (45, 46). Mauchly's test of sphericity was performed to test the assumption of equal variances of the differences between conditions. When the assumption was violated, degrees of freedom were adjusted using the Greenhouse–Geisser correction. The Mann–Whitney *U* Test was employed for non-normally distributed factors between groups. For Δ%(MF), Δ% (SMR power), and Δ% (Mu power), a *t*-test was implemented for those that exhibited a normal distribution, and the Wilcoxon Signed-Rank Test or Mann–Whitney *U* Test was employed for those that did not conform to a normal distribution. Multiple comparisons using a paired *t*-test with Bonferroni correction were used as a *post-hoc* test.

## 3 Result

### 3.1 Theta-band MF and change rate

**Figure 2:**
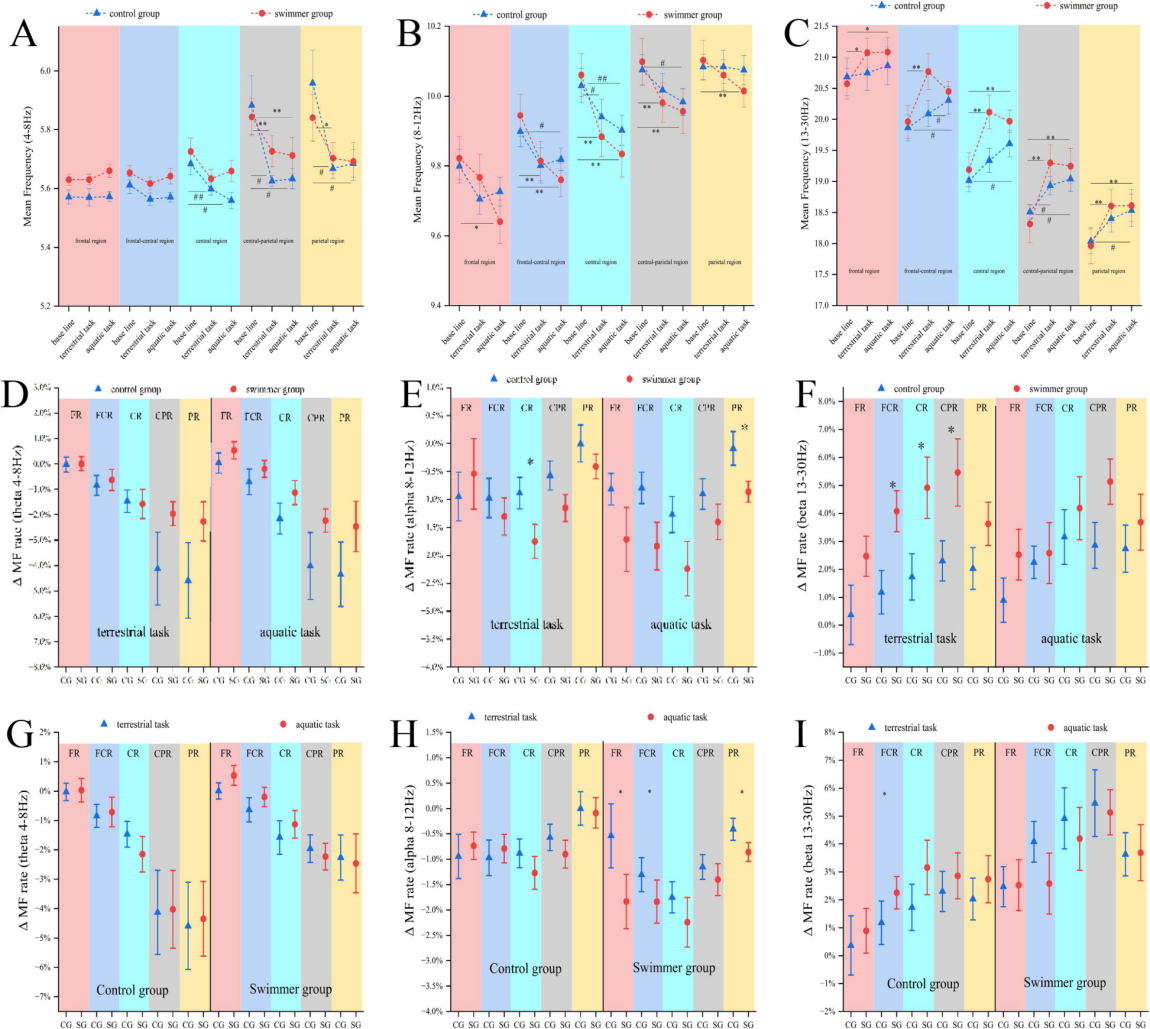
Mean frequency (MF) and the rate of change at theta, alpha, beta spectrum (mean ± 1SEM) on base line, terrestrial, aquatic conditions. **(A)** MF at theta. **(B)** MF at alpha. **(C)**: MF at beta. In the theta band, both groups demonstrated a general reduction in MF during task performance in both conditions relative to the resting baseline. In the alpha band, both groups exhibited a marked reduction in MF during tasks relative to the resting baseline. In the beta band, both groups exhibited significantly increased MF across all five regions. The rate of change between groups, **(D)** MF (theta), **(E)** MF (alpha), **(F)** MF (beta). The rate of change between task conditions, **(G)** MF (theta), **(H)** MF (alpha), **(I)** MF (beta). FR, frontal region (pink); FCR, frontal-central region (blue); CR, central region (cyan); CPR, central-parietal region (gray); PR, parietal region (yellow). * significant difference at SG, # significant difference at CG *p* ≤ 0.05; ** highly significant difference at SG, ## highly significant difference at CG *p* ≤ 0.01.

In the theta band, both groups exhibited a general trend of decreased MF during tasks in terrestrial and aquatic conditions compared to the resting state, as illustrated in Figure 2A, with partial statistical significance. Specifically, in the SG, MF during the task condition significantly decreased relative to baseline in the central-parietal region (*p* = .001) and parietal region (*p* = .020). In the CG, significant MF reductions were observed in the central (*p* = .002), central-parietal (*p* = .007), and parietal regions (*p* = .009). No significant group differences were found in MF change rates within the theta band (Figures 2D,G).

### 3.2 Alpha band MF and change rate

In the alpha band (Figure 2B), MF(SG) significantly decreased from baseline across all five regions: frontal (*p* = .009), frontal-central (*p* = .001), central (*p* = .001), central-parietal (*p* = .001), and parietal (*p* = .001). MF(CG) significantly decreased in the frontal-central (*p* = .016), central (*p* = .001), and central-parietal (*p* = .008) regions. About MF change rate, SG shows a significant decrease in an aquatic task at the frontal, frontal-central, and parietal regions compared with a terrestrial task. Compared with CG, the SG exhibited a significantly bigger MF change at the central region in terrestrial task, and also a significant bigger MF change at the parietal region in aquatic task (Figures 2E,H).

### 3.3 Beta band MF and change rate

In the beta band (Figure 2C), MF(SG) showed significantly increased MF in all five regions during both task conditions compared to resting baseline: frontal (*p* = .003), frontal-central (*p* = .002), central (*p* = .001), central-parietal (*p* = .001), and parietal (*p* = .001). MF(CG) significant increases were found only in selective regions: frontal-central (*p* = .018), central (*p* = .004), central-parietal (*p* = .002), and parietal (*p* = .007) in Figure 2C. Notably, the SG demonstrated significantly greater MF change rates than the CG during the land condition at the frontal-central (*p* = .012), central (*p* = .030), and central-parietal (*p* = .034) regions. Interestingly, MF(SG) in the aquatic task remained stable or even decreased relative to the terrestrial task, while MF(CG) in the aquatic task keeps slight increasing compared to a terrestrial task (Figures 2F,I).

### 3.4 Power ratio at theta, alpha and beta band

**Figure 3:**
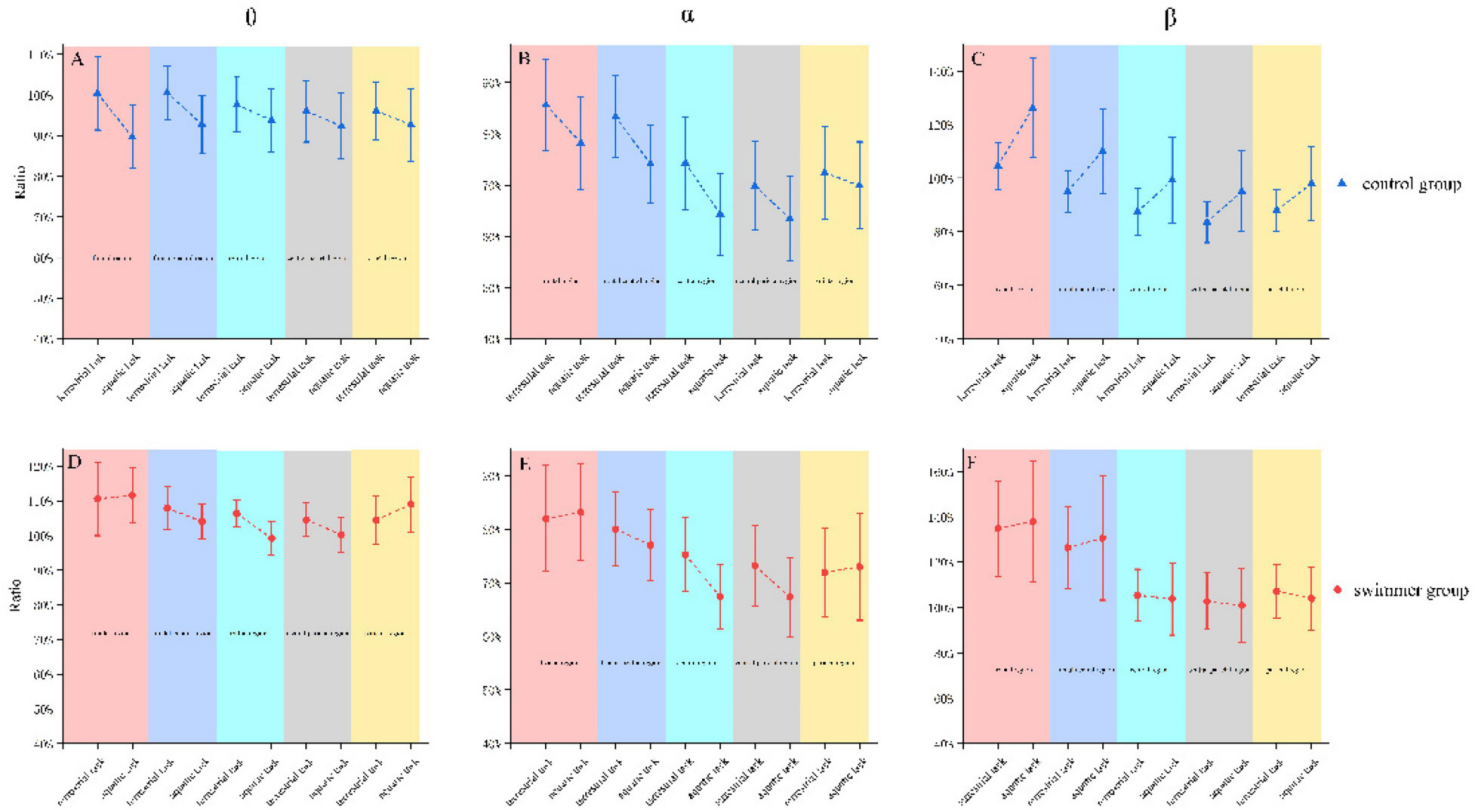
Power ratio(mean ± 1SEM) at theta, alpha, beta spectrum on terrestrial, aquatic tasks. In the theta band, power ratios were comparable between the aquatic and terrestrial conditions **(A,D)**. In the alpha band, control group shows lower power ratios across all cortical regions than the terrestrial task **(B)** Swimmer group mainly shows lower power ratios during the aquatic task than terrestrial over frontocentral, central, and central-parietal areas **(E)** In the beta band, control group showed higher power ratios in the aquatic than the terrestrial task across regions **(C)** Swimmer group power ratios were largely comparable between tasks **(F)**

In the theta band, power ratios were comparable between the aquatic and terrestrial conditions (Figures 3A,D). In the alpha band, task-related power was consistently lower than at rest. In the control group, power ratios during the aquatic task were lower than during the terrestrial task across all cortical regions (Figure 3B), whereas in the swimmer group lower aquatic than terrestrial power ratios were mainly observed over frontocentral, central, and central-parietal areas (Figure 3E). In the beta band, task-related power was consistently higher than at rest the control group showed higher power ratios in the aquatic than the terrestrial task across regions (Figure 3C), while in the swimmer group power ratios were largely comparable between tasks (Figure 3F). None of these trends reached statistical significance for either group or task condition in the theta, alpha, or beta bands.

### 3.5 SMR power and change rate at Cz

**Figure 4:**
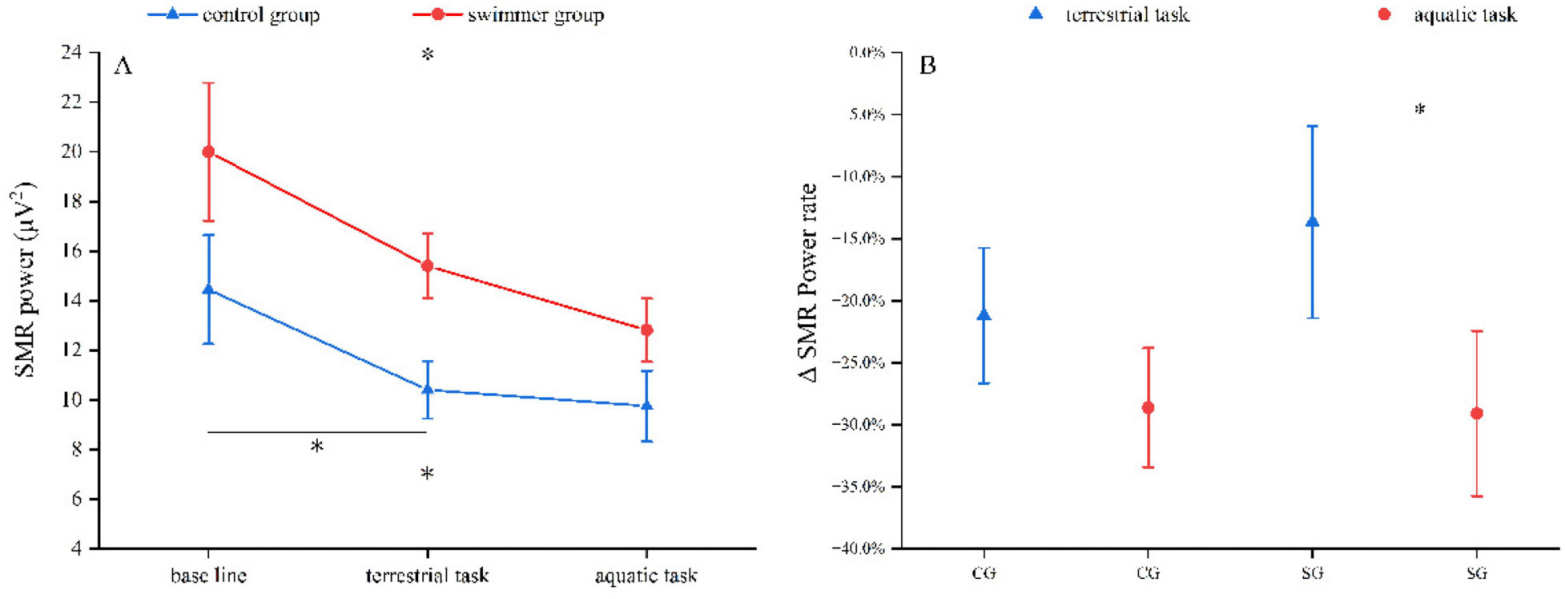
SMR power and ΔSMR power rate. **(A)** the power of SMR at Cz on different task conditions. **(B)** ΔSMR power rate at Cz on different task conditions. Both groups exhibited reduced SMR power during task conditions compared to baseline. *post-hoc* tests revealed that in the control group, SMR power significantly decreased in both terrestrial and aquatic tasks. In the swimmer group, a significant power decrease during the aquatic task. The ΔSMR power rate was significantly greater during the aquatic task than during the terrestrial task in the swimmer group. In contrast, the control group showed no significant difference in ΔSMR power rate between the two tasks. * Significant difference, *p* ≤ 0.05.

At the Cz electrode, both groups exhibited significantly reduced SMR power during task conditions compared to baseline (SG: *p* = .003; CG: *p* = .002), as showed in Figure 4A. *post-hoc* tests revealed that in the CG, SMR power significantly decreased in both terrestrial (*p* = .027) and aquatic (*p* = .013) environments. The SG exhibited a significant SMR power decrease exclusively during the aquatic environments (*p* = .019). Additionally, the SG displayed significantly lower SMR change rates when comparing aquatic to terrestrial conditions (*p* = .037) in Figure 4B.

The above sections summarize changes in mean frequency and power in the theta, alpha, beta, and SMR bands. Detailed statistics are provided in the Supplementary Materials (Supplementary Tables S1–S5).

## 4 Discussion

This study aimed to elucidate the neural mechanisms underlying cognitive-motor regulation in aquatic environments through an expert-novice paradigm, thereby revealing the neuroplastic foundations of performance differences between national level swimmers and non-athletes across aquatic and terrestrial settings. By comparing the dynamic changes in MF across cortical regions, SMR, band power at the Cz electrode during sensorimotor task execution in both environments, we found the following: (1) Swimmers exhibited higher SMR power during terrestrial tasks but showed a significant reduction in SMR power in the aquatic environment. (2) Distinct modulation patterns of MF and power were observed across frequency bands. In beta band Swimmers displayed either decreased or stable MF and power values during aquatic tasks compared to terrestrial tasks, whereas controls showed consistently higher MF and power values in the aquatic environment. These findings provide cortical function evidence—through the lens of neural oscillations—for the regulatory effects of long-term aquatic training on environmental adaptation, offering important implications for optimizing training protocols and advancing the theoretical framework of cross-environment skill transfer.

### 4.1 Mean frequency modulation and power ratio at different spectrum

MF provides a quantitative measure of cortical firing intensity proximal to the recording electrode (37, 47). Theta-band oscillations are closely associated with the coordinating functions of cognitive control and motor adaptation, serving as the foundational mechanism underlying various forms of action regulation (20). Such oscillations are also suited for integrating information across spatial domains (39). In this study, the control group exhibited significantly lower theta MF in the central, central-parietal, and parietal regions during task performance, while the swimmer group showed significant MF reductions in the central-parietal and parietal regions. These findings suggest coordinated engagement of motor-related regions (central and central-parietal) (48, 49) and somatosensory-related regions (parietal) (50, 51) in the execution of sensorimotor tasks (repetitive elbow flexion-extension). Theta oscillations are also implicated in working memory encoding processes (52). In our experiment, participants were first asked to memorize a target elbow angle, and then reproduce it as accurately as possible. The theta activity observed may reflect the encoding and retrieval processes of joint angle in task execution (53, 54).

In the alpha band, both groups showed lower MF and power spectrum during task conditions compared to the resting baseline. Significant MF decreases were observed in the swimmer group across all five regions (frontal, frontal-central, central, central-parietal, parietal), while the control group showed significant reductions in the frontal-central, central, and central-parietal regions. Reduced alpha-band MF is generally associated with cortical activation and the suppression of task-irrelevant activity during motor performance (21). These MF and power reductions likely reflect increased sensorimotor engagement during task execution. Notably, the swimmer group showed significantly bigger MF changes in an aquatic environment, especially in the frontal (FR), frontal-central (FCR), and parietal (PR) regions. Alpha-band event-related desynchronization (ERD) has been linked to attentional control, particularly in sharpening attentional focus and suppressing irrelevant information processing (55). These results suggest that swimmers may reduce interference from buoyancy and drag force in water, thereby facilitating improved motor performance in aquatic environments.

Physical exercise is commonly accompanied by beta-band cortical desynchronization (ERD) (23). However, a clear increase in beta-band mean frequency (MF) and power during movement was recorded in this research. We speculate that the task paradigm (periodic elbow flexion–extension) imposed an alternating start–stop structure, requiring participants to repeatedly and rapidly terminate ongoing actions, thereby amplifying beta oscillations. In recent research, transient post-movement increases in beta power—post-movement beta rebound (PMBR) or beta event-related synchronization (*β*-ERS)—can occur after each movement during repetitive actions (56). PMBR is typically observed after voluntary movement completion or following somatosensory stimulation (57, 58), and successful movement inhibition is likewise associated with increased beta activity (59, 60). PMBR generally peaks ∼0.5–1.0 s after movement offset and returns to baseline within ∼1 s, and has been reported in the sensorimotor cortices, premotor cortex, SMA, and medial prefrontal cortex (61–63). Accordingly, PMBR is widely considered a physiological marker of movement inhibition, reflecting active suppression within motor cortical networks following movement termination and/or sensory feedback (64).

In previous research, when participants were required to terminate more rapidly, PMBR was stronger immediately after movement termination (64). This likely reflects greater recruitment of executive control networks to rapidly terminate the movement (64). In other words, PMBR enhancement reflects increased demands on movement termination and inhibitory control. In our study, the control group showed a slight increase in beta-band MF and power during aquatic vs. terrestrial tasks, whereas swimmers exhibited stable or slightly reduced beta-band MF and power under aquatic conditions. This pattern may suggest that controls experience greater perturbations when maintaining arm position in water, increasing the demands on movement termination. However, swimmers may show greater resilience to aquatic perturbations.

Notably, interpreting enhanced beta activity during continuous elbow flexion–extension solely in terms of motor inhibition should not be the only explanation. Such complex movements likely involve mixed processes, potentially including working memory and attentional demands in addition to inhibitory control (59, 65). Future work should further examine alternative mechanisms that may contribute to the observed beta enhancement.

### 4.2 SMR power and change rate with the cortical activation

In the swimmer group, SMR power at Cz during terrestrial tasks were significantly diminished compared to baseline, and the ΔSMR% during aquatic tasks were significantly greater than during terrestrial tasks. These observations indicate that the sensorimotor cortex of swimmers exhibited reduced activation on land tasks but demonstrated enhanced neural engagement in aquatic environments. Given the inherent buoyancy and drag force in water, the observed enhanced activation likely reflects increased sensorimotor processing demands in response to environmental perturbations. Notably, this pattern was absent in the control group, which demonstrated comparable levels of cortical activation across different environmental conditions. Pianists exhibited significantly lower alpha-band power during complex vs. simple keypress tasks, whereas non-pianists showed no significant task-condition differentiation (12). This research indicates that both task complexity and motor expertise shape cortical activation patterns during task execution. In our study, SMR power modulation (12–15 Hz) across environmental conditions appears to reflect the interactive effects of environmental interference and motor expertise on sensorimotor control. Previous studies have consistently linked elevated SMR power with reduced somatosensory interference (66), improved attentional performance, and optimized motor output (67). Our result suggests that long-term swim training may facilitate SMR-related cortical plasticity, consequently enhancing motor performance in aquatic environments.

Overall, controlling limb movements in water entails greater external interference than in a terrestrial environment. During aquatic movement, both swimmers and controls likely require additional cognitive control, sustained attentional focus, and suppression of task-irrelevant processing, accompanied by heightened task-related activation in the relevant cortical regions. Notably, the two groups also exhibited different levels of environmental adaptation. In swimmers, enhanced engagement of water-specific sensorimotor processing was reflected by a further reduction in SMR power. In contrast, controls may have recruited executive control networks to a greater extent to implement active inhibition in water, consistent with the broadly increased beta activity observed across regions during movement.

## 5 Limitations and future directions

Although this study provides insights into the impact of training and environmental conditions on EEG activity through multiple neurophysiological indices, several limitations should be acknowledged. First, despite our experimental design using a water tank that precisely controlled hydrostatic pressure and temperature, participants' range of motion. Consequently, the experimental conditions may not fully replicate the neural dynamics involved in realistic swimming tasks that require multi-joint coordination and whole-body movement. Future studies should employ more complex, multi-joint paradigms (e.g., simulated swimming stroke tasks) to enhance ecological validity. Second, the relatively small sample size in this study may limit the generalizability and statistical power of the results. Future research with larger samples is necessary to improve the robustness and reliability of the findings. Third, the present investigation did not include swimmers of varying competitive levels (e.g., amateur vs. elite). Consequently, potential relationships between neural oscillation patterns and motor performance metrics (such as movement accuracy and force output efficiency) remain unexplored. Future research could integrate kinematic parameters (e.g., joint trajectory analysis) with functional connectivity analyses of brain networks, investigating cross-frequency oscillatory interactions that underpin motor control.

## 6 Conclusion

To our knowledge, this is the first EEG study to compare swimmers and non-athletes in cortical activity during aquatic vs. terrestrial motor tasks. During continuous elbow flexion–extension, we observed that periodic movement termination may markedly amplify beta activity. Compared with the terrestrial condition, non-athletes showed stronger beta activity in water, suggesting that stopping movement under aquatic conditions demands greater neural processing. In swimmers, the SMR power change (ΔSMR power) was significantly larger in water than on land, indicating environment-specific engagement of the sensorimotor cortex. Collectively, these results suggest that swimmers may better tolerate aquatic perturbations by flexibly modulating sensorimotor cortical activation, offering new insight into how neural oscillations relate to motor performance and highlighting potential neurophysiological targets for training optimization.

## Data Availability

The raw data supporting the conclusions of this article will be made available by the authors, without undue reservation.
