# Supplementary material for: Cortical inhibition and activation during sensorimotor tasks in an aquatic environment: a pilot EEG study based on expert-novice paradigm
Source: Front Sports Act Living. 2026 Feb 10;8:1660332. doi: 10.3389/fspor.2026.1660332 (PMC12928708; doi:10.3389/fspor.2026.1660332)
Supplement: Supplementary file 1 [file Presentation1.pdf]

SUPPLEMENTARY MATERIALS

control group

Base line | Terrestrial condition | Aquatic condition | PSD(dB)

theta(4-8hz)

alpha(8-12hz)

beta(13-30hz)

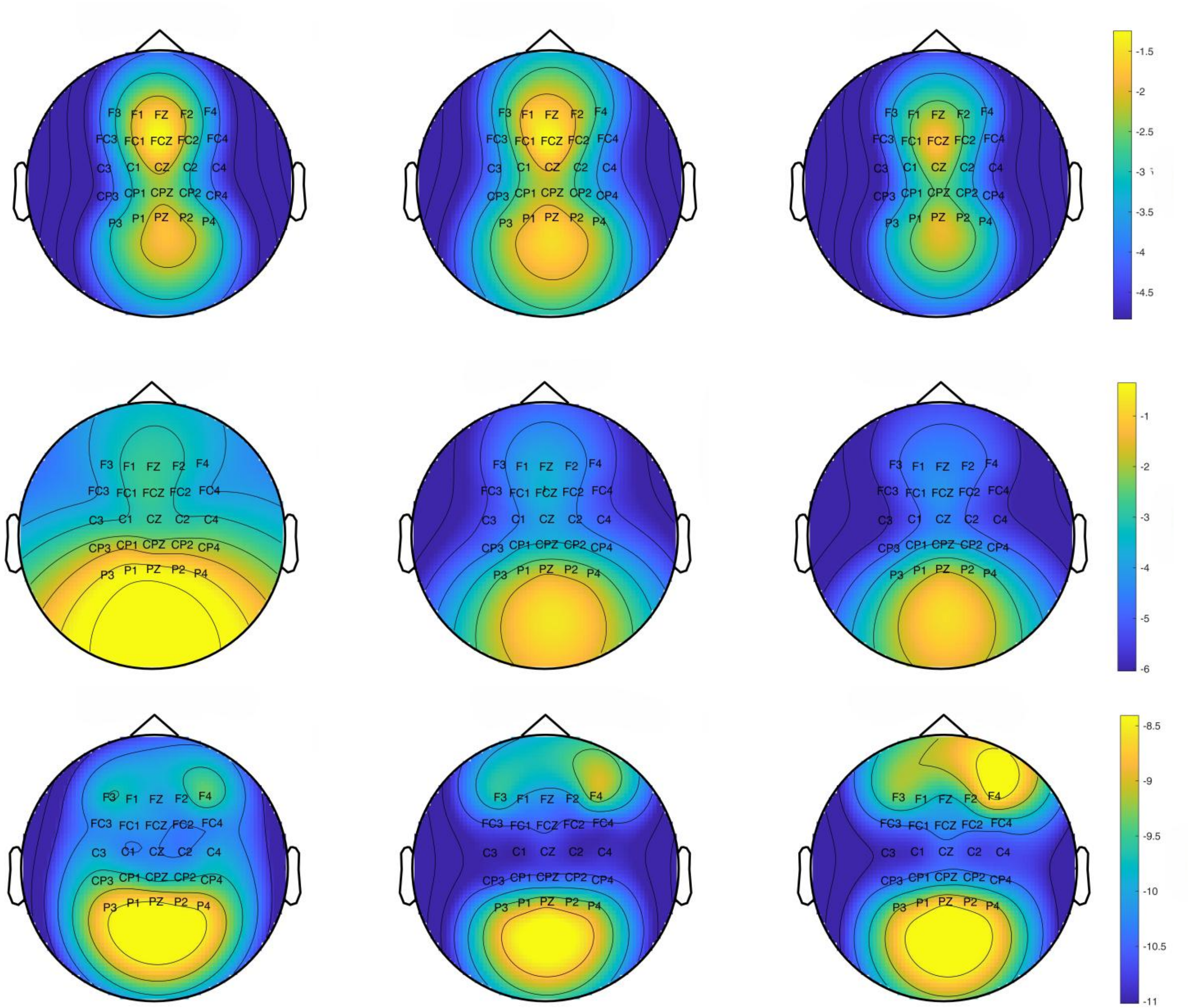

Fig. S1. Power spectral density (PSD, dB) of the control group across brain regions and frequency bands (theta, alpha, beta) during the base line, terrestrial condition and aquatic condition.

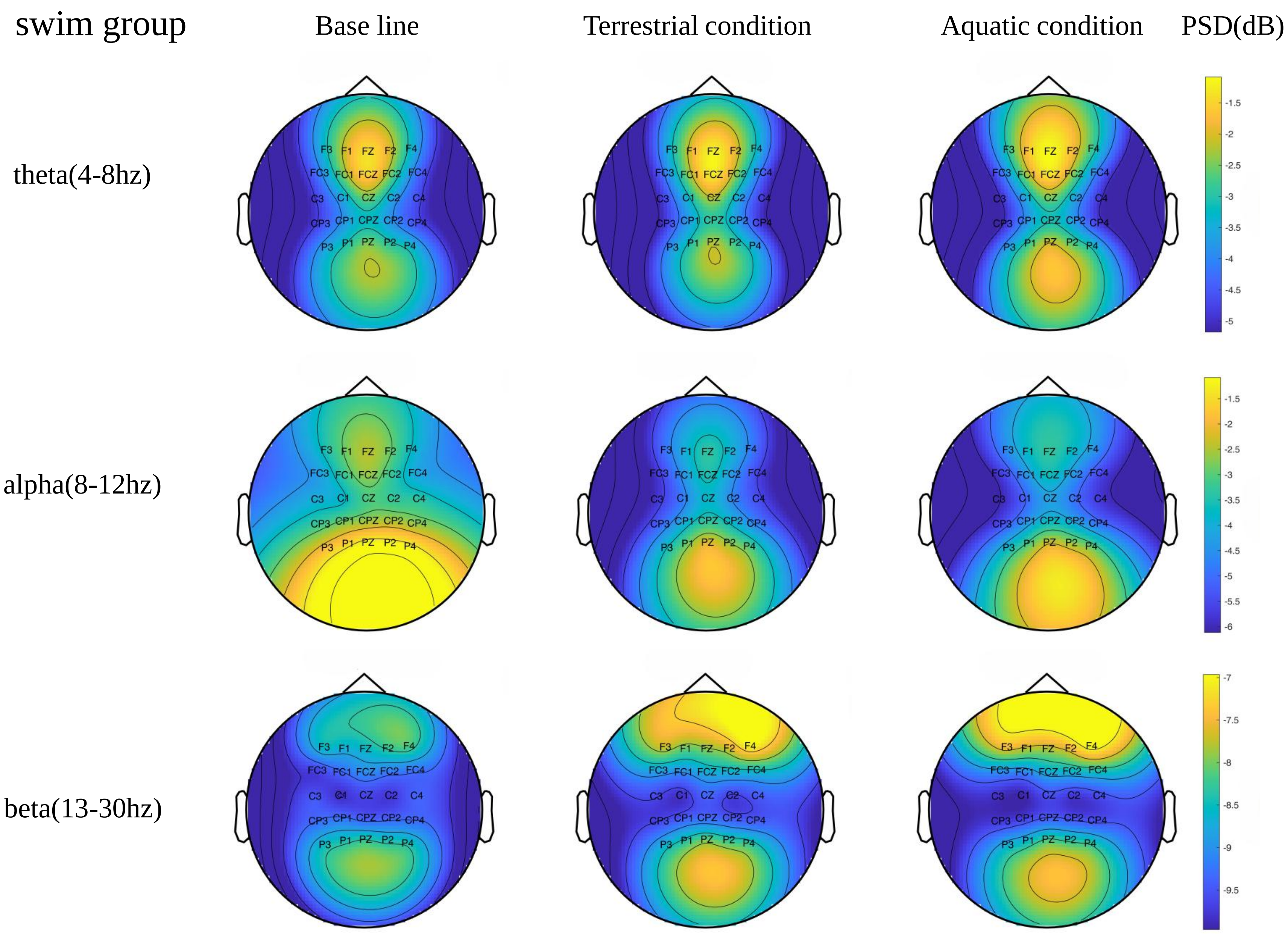

Fig. S2. Power spectral density (PSD, dB) of the swim group across brain regions and frequency bands (theta, alpha, beta) during the base line, terrestrial condition and aquatic condition.

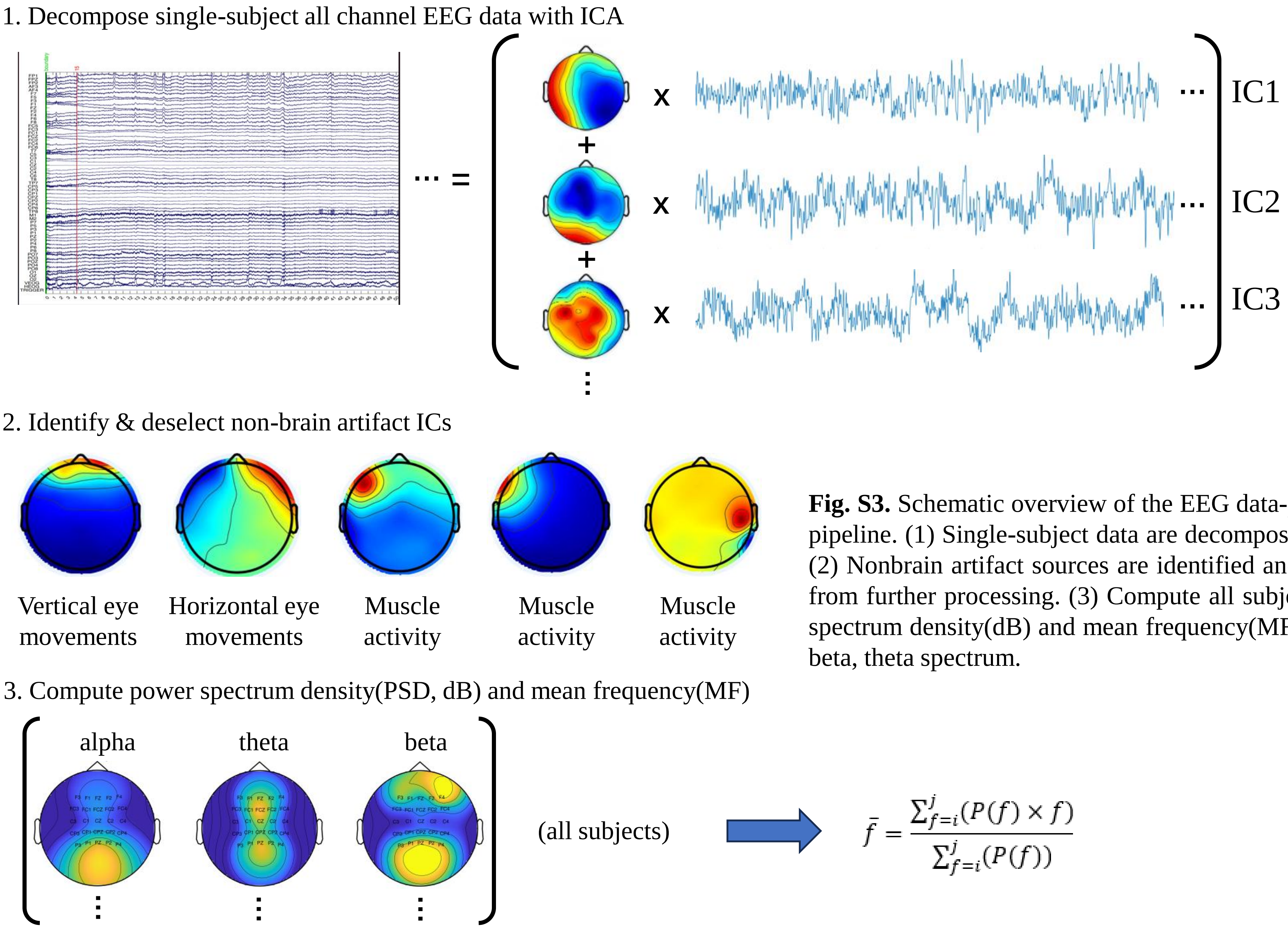

Fig. S3. Schematic overview of the EEG data-processing pipeline. (1) Single-subject data are decomposed by ICA. (2) Nonbrain artifact sources are identified and removed from further processing. (3) Compute all subjects power spectrum density(dB) and mean frequency(MF) at alpha, beta, theta spectrum.

Table. S1. Comparison of mean frequency(MF) among conditions

Frontal region

		base line	terrestrial task	aquatic task	F	p	η^2
θ	Control	18.667±11.412	17.167±12.54	19.667±7.887	0.398	0.676	0.035
	Swimmer	5.63±0.065	5.63±0.062	5.66±0.078	1.791	0.194	0.14
α	Control	9.799±0.168	9.705±0.151	9.726±0.143	3.07	0.067	0.218
	Swimmer	9.822±0.212	9.767±0.227	9.64±0.215[a]	5.905	0.009	0.349
β	Control	17.25±11.096	18.25±10.83	20±10.419	0.625	0.544	0.054
	Swimmer	20.576±0.852	21.076±0.803[a]	21.084±0.802[a]	7.773	0.003	0.414

Frontal-central region

		base line	terrestrial task	aquatic task	F	p	η^2
θ	Control	21.75±11.741	15.75±11.096	18±8.493	2.095	0.147	0.16
	Swimmer	5.653±0.085	5.617±0.075	5.642±0.091	1.307	0.291	0.106
α	Control	9.898±0.15	9.801±0.176	9.819±0.111[a]	5.055	0.016	0.315
	Swimmer	9.944±0.209	9.814±0.195[b]	9.76±0.166[b]	15.48	0.001	0.585
β	Control	15±11.273	18.17±10.744[c]	22.33±9.008[a]	6.165	0.007	0.359
	Swimmer	19.96±0.931	20.77±1[b]	20.45±0.557	8.151	0.002	0.426

Central region

		base line	terrestrial task	aquatic task	F	p	η^2
θ	Control	25.08±8.929	16.58±9.624[b]	13.83±10.285[a]	8.461	0.002	0.435
	Swimmer	5.726±0.157	5.633±0.107	5.659±0.123[a]	5.905	0.009	0.349
α	Control	10.03±0.169	9.941±0.177[a]	9.902±0.15[b]	9.227	0.001	0.456
	Swimmer	10.06±0.213	9.883±0.196[b]	9.834±0.228[b]	18.948	0.001	0.633
β	Control	19.014±0.637	19.337±0.667	19.607±0.741[a]	7.257	0.004	0.397
	Swimmer	11.17±10.794	22.08±9.539[b]	22.25±7.593[b]	11.906	0.001	0.52

Central-parietal region

		base line	terrestrial task	aquatic task	F	p	η^2
θ	Control	24.167±10.845	15±8.043[a]	16.333±10.832[a]	6.342	0.007	0.366
	Swimmer	5.843±0.223	5.727±0.182[b]	5.712±0.214[b]	17.3	0.001	0.611
α	Control	10.075±0.152	10.017±0.165	9.984±0.134[a]	6.058	0.008	0.355
	Swimmer	10.099±0.229	9.981±0.197[b]	9.956±0.219[b]	14.379	0.001	0.567
β	Control	18.509±0.418	18.932±0.515[a]	19.039±0.691[a]	8.57	0.002	0.438
	Swimmer	18.317±1.05	19.299±0.999[b]	19.245±0.983[b]	24.328	0.001	0.689

Parietal region

		base line	terrestrial task	aquatic task	F	p	η^2
θ	Control	5.959±0.389	5.668±0.118[a]	5.685±0.163[a]	9.167	0.009	0.455
	Swimmer	5.841±0.277	5.703±0.182[a]	5.692±0.223	6.599	0.02	0.375
α	Control	19.75±10.376	17.92±12.026	17.83±9.916	0.333	0.72	0.029
	Swimmer	10.103±0.198	10.06±0.152	10.015±0.159[b]	9.53	0.001	0.464
β	Control	18.039±0.691	18.401±0.746	18.534±0.902[a]	6.222	0.007	0.361
	Swimmer	17.966±1.027	18.605±0.935[b]	18.611±0.901[b]	12.841	0.001	0.539

a: compare with base line p<0.05; b: compare with base line p<0.01; c: compare with swimmer group p<0.05; Post-hoc analysis have been validated by Bonferroni correction.

Table.S2. Between-group comparisons of ΔMF(mean frequency) rate in cortical regions

task	spectrum	cortical region	control	swimmer	t	p
terrestrial	α	central	-0.887±0.977	-1.751±1.056	2.079	0.049
aquatic		parietal	-0.088±1.048	-0.861±0.64	2.181	0.04
terrestrial	β	frontal-central	1.181±2.685	4.08±2.533	-2.721	0.012
		central	1.731±2.861	4.917±3.804	-2.318	0.03
		central-parietal	2.029±2.591	3.626±2.676	-2.263	0.034

Table.S3. Within-group comparisons of ΔMF(mean frequency) rate between terrestrial and aquatic task

group	spectrum	cortical region	terrestrial task	aquatic task	t/z	p
swimmer	α	frontal	-0.54±2.193	-1.833±1.85	2.684	0.021
		frontal-central	-1.305±1.16	-1.836±1.469	2.254	0.046
		parietal	-0.217(0.921)	-0.789(0.732)	2.118	0.034
control	β	frontal-central	1.181±2.685	2.253±2.01	-2.469	0.031

Table.S4. Comparison of sensorimotor rhythm(SMR) power among conditions

	base line	terrestrial task	aquatic task	F	p	η^2
control	14.453±7.612	10.413±4.015[a]	9.756±4.951[a]	8.608	0.002	0.439
swimmer	20.007±9.615	15.409±4.514	12.819±4.413[a]	7.588	0.003	0.408

a: compare with base line p<0.05; Post-hoc analysis have been validated by Bonferroni correction.

Table.S5. Within-group comparisons of ΔSMR rate between terrestrial and aquatic task

group	terrestrial task	aquatic task	t	p
swimmer	-13.65±26.8	-29.077±23.067	2.375	0.037

Given the large volume of data, only statistically significant results are presented.
